# Risk factors associated with prolonged hospital length-of-stay: 18-year retrospective study of hospitalizations in a tertiary healthcare center in Mexico

**DOI:** 10.1371/journal.pone.0207203

**Published:** 2018-11-08

**Authors:** Braulio A. Marfil-Garza, Pablo F. Belaunzarán-Zamudio, Alfonso Gulias-Herrero, Antonio Camiro Zuñiga, Yanink Caro-Vega, David Kershenobich-Stalnikowitz, José Sifuentes-Osornio

**Affiliations:** 1 Instituto Nacional de Ciencias Médicas y Nutrición Salvador Zubirán, Mexico City, Mexico; 2 Departamento de Infectología, Instituto Nacional de Ciencias Médicas y Nutrición Salvador Zubirán, Mexico City, Mexico; Medical University Graz, AUSTRIA

## Abstract

**Background:**

Hospital length-of-Stay has been traditionally used as a surrogate to evaluate healthcare efficiency, as well as hospital resource utilization. Prolonged Length-of-stay (PLOS) is associated with increased mortality and other poor outcomes. Additionally, these patients represent a significant economic problem on public health systems and their families. We sought to describe and compare characteristics of patients with Normal hospital Length-of-Stay (NLOS) and PLOS to identify sociodemographic and disease-specific factors associated with PLOS in a tertiary care institution that attends adults with complicated diseases from all over Mexico.

**Materials and methods:**

We conducted a retrospective analysis of hospital discharges from January 2000-December 2017 using institutional databases of medical records. We compared NLOS and PLOS using descriptive and inferential statistics. PLOS were defined as those above the 95^th^ percentile of length of hospitalization.

**Results:**

We analyzed 85,904 hospitalizations (1,069,875 bed-days), of which 4,427 (5.1%) were PLOS (247,428 bed-days, 23.1% of total bed-days). Hematological neoplasms were the most common discharge diagnosis and surgery of the small bowel was the most common type of surgery. Younger age, male gender, a lower physician-to-patient ratio, emergency and weekend admissions, surgery, the number of comorbidities, residence outside Mexico City and lower socioeconomic status were associated with PLOS. Bone marrow transplant (OR 18.39 [95% CI 12.50–27.05, *p*<0.001), complex infectious diseases such as systemic mycoses and parasitoses (OR 4.65 [95% CI 3.40–6.63, *p<*0.001), and complex abdominal diseases such as intestinal fistula (OR 2.57 [95% CI 1.98–3.32) had the greatest risk for PLOS. Risk of mortality in patients with PLOS increased more than threefold (3.7% vs 13.3%, p<0.001).

**Conclusions:**

We report some key sociodemographic and disease-specific differences in patients with PLOS. These could serve to develop a specific model of directed hospital healthcare for patients identified as in risk of PLOS.

## Introduction

In 2014, 44% of Mexicans’ health expenditure was absorbed by patients themselves, compared to 11% in the U.S.A.[1]. Such difference highlights the need for strategies to control health expenditure in Mexico and to evaluate healthcare efficiency and resource utilization, particularly, concerning health expenditure on hospitalized patients. Hospital Length-Of-Stay (LOS) refers to the total bed-days occupied by a patient during his hospitalization, and it has been used as a traditional surrogate to evaluate efficiency of healthcare, effectiveness of preventive and therapeutic strategies, diagnostic methods, clinical pathways, as well as hospital resource utilization, allocation, and administration[2]. The operational indicator for hospital LOS is the average length-of-stay, and by this measure patients may be classified as those with a Normal Length-Of-Stay (NLOS) and those with a Prolonged Length-Of-Stay (PLOS). Although the latter term has not been standardized, overall, these patients have worse outcomes, both from the health and socioeconomic perspectives[3–5]. Moreover, despite the relevance of LOS in healthcare administration and healthcare epidemiology, there is a lack of knowledge about factors related to LOS in Mexico and Latin America. Hence, we sought to describe the frequency of PLOS in a tertiary healthcare referral center located in Mexico City, analyze changes in LOS through time, describe the characteristics of the events of hospitalization associated to PLOS, and identify factors associated with this outcome.

## Materials and methods

### Study setting and design

We analyzed our Institution´s hospital discharge database which contains information of all hospitalization events. The study derived from an institutional monitoring program to assess LOS during 2016 and was later extended as a retrospective, cross-sectional analysis of all episodes of hospitalization from January 2000 to December 2017. Our hospital, a public tertiary healthcare referral center located in Mexico City, is one of the Mexican National Institutes of Health (MNIH) and provides healthcare to adult patients with complex diseases from all over the country. At this institution there are 167 hospital beds available for admission, and the annual average of hospitalization events is 4772.4 (SD ± 346.6).

### Case definition and outcomes

Only events that included at least one day of stay in the general hospital wards during their total hospitalization were included in the analysis. We excluded hospitalization events that were exclusively managed in the Emergency Department [ED], in the Intensive Care Unit [ICU] or both (N = 5,441). LOS was considered from the day of hospital admission (regardless of the initial service of admission) to the day of hospital discharge or death (regardless of the service of discharge or death). During each event of hospitalization, patients could be transferred several times to different areas of the hospital depending on their clinical status (e.g. from the wards to the ICU and back to the wards). The main diagnosis at hospital discharge or death, was considered the reason for hospitalization.

We defined PLOS events based on the 95^th^ percentile LOS, which has been previously used [4]. In our institution, that corresponded to ≥34 days. NLOS patients were defined as those with a LOS <34 days. The following variables were evaluated for all episodes of hospitalization: LOS in days, age at discharge in years, gender, type of admission (elective or emergency), type of hospitalization (as previously described[6]; “elective, non-surgical”, “elective, surgical”, “emergency, non-surgical”, and “emergency, surgical”), type of hospital bed (shared or private), physician-to-patient ratio (20 beds per medical team [January 2000-February 2008] or 12 beds per medical team [March 2008-December 2017]), day of admission (weekday [Monday-Thursday] or weekend [Friday-Sunday]), total number of events of hospitalization, number and type of readmission (early: ≤30 days from a previous hospitalization event or late: >30 days from a previous hospitalization event), days to readmission, surgery (only those procedures occurring in operating rooms), number of surgeries during hospitalization event, number of additional diagnoses (comorbidities), place of residence, diagnosis at discharge, socioeconomic status, in-hospital crude mortality and location of death (hospitalization ward, ED or ICU).

Socioeconomic status is a construct used by the MNIH that comprises the following elements: monthly household income, family’s main provider’s occupation, monthly household expenses, housing conditions and family’s health status. By this measure, patients are classified in seven levels (1–7) which are inversely related to the magnitude of subsidy over health expenditure during hospitalization. The International Classification of Diseases, in its 9th version was used for codification of surgeries and its 10th version for diagnoses. Diagnoses and surgeries were classified in groups for analysis (55 diagnostic groups and 30 surgical groups). These groups were organized and agreed by all authors considering frequency of the disease or surgery and specific clinical characteristics (e.g. diagnosis, prognosis, and treatment)(S1 and S2 Tables). This classification is similar to others previously published and validated [7].

### Statistical analysis

A descriptive and comparative analysis of PLOS and NLOS patients was conducted. Quantitative variables were compared with a Student’s *t* test or a Mann-Whitney U test, according to their distribution after applying skewness and kurtosis tests for normality. Categorical variables were compared using the chi-squared test. We used multivariate logistic regression analysis to identify factors associated with PLOS. First, to identify the risk of PLOS by group of diagnosis adjusting for potential confounders, we fit a multinomial logistic regression model for the 55 different diagnostic groups. We included age, gender, physician-to-patient ratio, type of admission, readmission at 30 days, day of admission (weekday vs weekend), number of additional diagnoses, place of residence and socioeconomic status. We used as reference “*Diseases of the liver*, *biliary tract and pancreas (K70*.*0-K79*.*9*, *K83*.*0-K89*.*9)*”, because it was the second largest diagnosis group (N = 6,426) and patients with PLOS and NLOS were equally distributed within this diagnosis group (7.7 vs 7.6%, respectively, *p =* 0.31). We used multiple chained equations to impute missing data with 10 imputations. Separately, to identify sociodemographic and clinical factors associated to PLOS controlling for the diagnosis of discharge, we fit a multinomial logistic regression model for PLOS based on the 55 different diagnostic groups. We generated inverse weights using the predictions of this univariate multinomial model for PLOS. We then fit a multivariate logistic regression model to estimate the risk of PLOS including age, gender, physician-to-patient ratio, type of admission, rehospitalization at 30 days, day of admission (weekday vs weekend), number of additional diagnosis, place of residence and socioeconomic status, and used inverse probability weights (IPW) to adjust for the effect of the discharge diagnosis on LOS. Missing data was handled as described above. We used Stata v12 software (StataCorp, 2012, College Station, Texas) for all statistical analyses.

### Ethical considerations and reporting

Every hospitalization episode was associated to an individual patient’s institutional registry number. These were used to combine patient transfers within the different areas of our hospital (wards, ICU, ED) into a single continuous episode. All data were de-identified and fully anonymized before the analysis. No informed consent was deemed necessary by our institution’s Ethics and Scientific Committees (Institutional Review Board) and the manuscript was evaluated and approved by these committees prior to submission for publication. We abide to the Principles of the Declaration of Helsinki.

## Results

### Description of hospitalization events

We analyzed 85,904 hospitalization events (1,069,875 bed-days) during the 18-year study period. We identified 4,427 PLOS events (5.1%) which corresponded to 23.1% of the total bed-days (247,428). The median hospital LOS for all events was 8 days (interquartile range [IQR] 5–14); 8 days for NLOS (IQR 5–13) and 45 days for PLOS (IQR 38–60). Median age at hospitalization was 51-years old (yo) (IQR 35–66). The median socioeconomic level was 3 [IQR 2–4]. Surgery was performed in 41.8% of hospitalization events. Overall, in-hospital crude mortality was 4.2% (n = 3,623).

### Characteristics of hospitalization events by length-of-stay

We compared the characteristics of hospitalization events by LOS (NLOS vs PLOS) in Table 1. Briefly, PLOS events occurred among younger people (48 years [IQR 32–62] vs 52 years [IQR 35–66], *p<*0.001); and had a lower median socioeconomic level (2 [IQR 2–3] vs 3 [IQR 2–4], *p<*0.001). When compared to NLOS, events of PLOS showed a greater proportion of admissions from the ED (28.8% vs 11.0%, *p*<0.001), were more likely to be admitted on weekends (36.5 vs 30.9%, *p<*0.001), and were more likely to occur in shared rooms (78.1 vs 71.9%, *p<*0.001). Surgeries occurred in 62.5% of PLOS hospitalizations and in 40.7% of NLOS (*p*<0.001). Also, PLOS hospitalizations were associated with a higher in-hospital crude mortality (13.3% *vs* 3.7%, *p<*0.001). Most of these deaths occurred in the hospital ward, but a greater proportion of patients with PLOS died in the ICU in comparison with NLOS patients (35.0 vs 18.9%, *p<*0.001). PLOS hospitalizations occurred as early readmissions (≤30 days) more frequently than NLOS hospitalizations (33.6 vs 27.9%, *p<*0.001). Moreover, the median time for readmission was shorter in PLOS hospitalizations, both for early (11 vs 13 days, *p*<0.001) and late (201 vs 261 days, *p<*0.001) readmissions.

**Table 1:** Comparison of characteristics of hospitalizations by type of episode (normal vs prolonged length-of-stay) from 2000–2017.

**Characteristic**	Normal length-of-stay (NLOS) (N = 81,477, 95%)	Prolonged length-of-stay (PLOS) (N = 4,427, 5%)	*p*
**Length-of-stay in days^a^**	8 (5–13)	45 (38–60)	*<*0.001
**Age at hospitalization**	52 (35–66)	48 (32–62)	<0.001
**Female (%)**	45,431 (55.76)	2,340 (52.9)	<0.001
**Residence (%)**			
• **Mexico City** • **Outside Mexico City** • **Unknown**	32,973 (40.5) 30,227 (37.1) 18,277 (22.4)	1,481 (33.5) 1,825 (41.2) 1,121 (25.3)	<0.001
**Type of admission (%)**			
• **Urgent** • **Elective**	8,939 (11.0) 72,538 (89.0)	1,277 (28.8) 3,150 (71.2)	<0.001
**Surgical patients (%)**	33,143 (40.7)	2,767 (62.5)	<0.001
**Shared Room (%)**	58,584 (71.9)	3,458 (78.1)	<0.001
**Patient to physician ratio (%)**			
• **20 patients per medical team (January 2000-February 2008)**	35,785 (43.9)	1,869 (42.2)	0.026
ਁ• **2 patients per medical team (March 2008-December 2017)**	45,692 (56.1)	2,558 (57.8)	
**Day of admission (%)**			
• **Weekday (Monday-Thursday)** • **Weekend (Friday-Sunday)**	56,329 (69.1) 25,148 (30.9)	2,842 (64.1) 1,591 (35.9)	<0.001
**Hospital readmissions (%)**	35,656 (43.8)	1,799 (40.6)	<0.001
**Distribution by type of readmission (%)**			
• **≤ 30 days** >• **30 days**	9,937 (27.9) 25,719 (72.1)	605 (33.6) 1,194 (66.4)	<0.001
**Time for readmission**			
• **≤ 30 days** • **> 30 days**	13 (7–21) 261 (93–793)	11 (6–18) 201 (81–713)	<0.001
**Surgeries per patient^b^**	1 (1)	2 (1–3)	<0.001
**Additional diagnoses**	3 (2–5)	5 (3–8)	<0.001
**Socioeconomic level**	3 (2–4)	2 (2–3)	<0.001
**Socioeconomic status^c^ (%)**			
• **Low (1–2)** • **Mid (3–4)** • **High (5–7)**	32,056 (39.3) 38,341 (47.1) 11,080 (13.6)	2,358 (53.3) 1,622 (36.6) 447 (10.1)	<0.001
**In-hospital Mortality (%)**	3,035 (3.7)	588 (13.3)	<0.001
**Location of death (%)**			
• **Hospital ward** • **Emergency Department** • **Intensive care unit**	1,887 (62.2) 574 (18.9) 574 (18.9)	272 (46.3) 110 (18.7) 206 (35.0)	<0.001

^a^ Continuous variables are summarized using medians and interquartile ranges (IQR)

^b^ Only surgical patients were included in this analysis (N = 35,910)

^c^ Socioeconomic status is a construct used by the MNIH that comprises the following elements: monthly household income, family`s main provider`s occupation, monthly household expenses, type of housing and family`s health status. Patients are classified in seven levels (1–7) and that determines the amount the patient should pay for healthcare.

**Fig 1:**
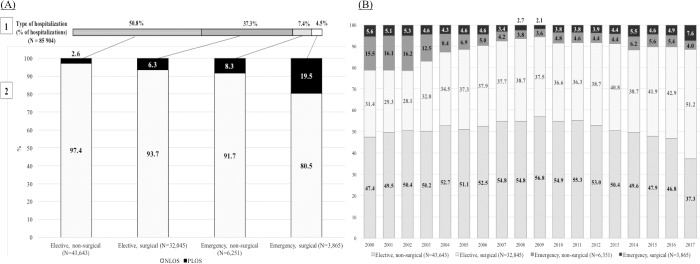
Distribution of prolonged length of stay (PLOS) events by type of hospitalization from 2000–2017. (A) Overall Distribution of PLOS events by type of hospitalization (elective or emergency and surgical and non-surgical). The frequency of PLOS was significantly higher during hospitalization events that required a surgical intervention. (B) Annual trends of the distribution of type of hospitalization. During the study period there was an important reduction in the proportion of elective and urgent surgical events of hospitalization (dark and light gray bars). Elective hospitalization events for surgical procedures increased the most during the study period.

In Fig 1 we show the hospitalization events according to four types of hospitalization, most hospitalizations were elective admissions with no surgical interventions performed during the hospitalization (“elective, non-surgical”, 50.8%); followed by elective admissions with surgeries performed during the hospitalization event (“elective, surgical”, 37.3%), emergency admissions with no surgeries performed during the hospitalization (“emergency, non-surgical”, 7.4%); and only a small proportion of hospitalizations were classified as “emergency, surgical”, 4.5%)(Fig 1, Panel A). While this latter group was smaller, it had the greatest proportion of hospitalizations with PLOS (19.5%) (Fig 1, Panel A).

### Diagnosis at hospital discharge

The twenty most common diagnoses are shown in Table 2. These comprise around 70% of all the hospitalizations, but their distribution differ by groups: 61.9% in PLOS and 72.9% in NLOS; thus, other less frequent diagnoses account for a greater proportion of PLOS events. **“***Malignant neoplasms of lymphoid*, *hematopoietic and related tissue (C81*.*0-C96*.*9)*” were the most common diagnosis in both populations.

**Table 2:** Frequency of the most common diagnoses at discharge and distribution according to length-of-stay from 2000–2017.

	DISEASE GROUP^a^	All events N (%)	NLOS^b^ N (% of row)	PLOS^b^ N (% of row)
**1**	Malignant neoplasms of lymphoid, hematopoietic and related tissue	6,602 (7.7)	6,127 (92.8)	475 (7.2)
**2**	Diseases of the liver, biliary tract and pancreas	6,426 (7.5)	6,097 (94.9)	329 (5.1)
**3**	Malignant neoplasms of digestive organs (oral cavity to anus)	5,517 (6.4)	5,214 (94.5)	303 (5.5)
**4**	Acute lung and upper and lower airway disease	4,561 (5.3)	4,185 (91.8)	376 (8.2)
**5**	Other unspecified renal diseases (e.g. glomerular, interstitial, etc.)	3,999 (4.7)	3,935 (98.4)	64 (1.6)
**7**	Common surgical procedures (appendectomy, hernia repair, cholecystectomy)	3,871 (4.5)	3,815 (98.6)	56 (2.4)
**6**	Other unspecified healthcare and contact with health services (e.g. trauma, burns, dependency on ventilators and other devices, etc.)	3,787 (4.4)	3,657 (96.6)	130 (3.4)
**8**	Ill-defined, secondary and of uncertain behavior malignant neoplasms	3,213 (3.7)	3,112 (96.9)	101 (3.1)
**9**	Arthropathies, dorsopathies, osteopathies and chondropathies	2,978 (3.5)	2,829 (95.0)	149 (5.0)
**10**	Other unspecified digestive diseases (including intestinal obstruction, functional disorders, GI bleeding, etc.)	2,655 (3.1)	2,547 (95.9)	108 (4.1)
**11**	Malignant neoplasms of male and female genital organs (including breast)	2,621 (3.1)	2,585 (98.6)	36 (1.4)
**12**	Other endocrine diseases and metabolic disorders (e.g. obesity, dyslipidemia, malnutrition, etc.)	2,235 (2.6)	2,167 (97.0)	68 (3.0)
**13**	Acute kidney failure and chronic kidney disease	1,904 (2.2)	1,840 (96.6)	64 (3.4)
**14**	Systemic connective tissue disorders	1,858 (2.2)	1,704 (91.7)	154 (8.3)
**15**	Other cardiovascular diseases (including rheumatic and pulmonary heart disease)	1,852 (2.2)	1,737 (93.8)	115 (6.2)
**16**	Diabetes mellitus and other disorders of glucose metabolism	1,696 (2.0)	1,635 (96.4)	61 (3.6)
**17**	Unspecified disorders of the circulatory system	1,641 (1.9)	1,599 (97.4)	42 (2.6)
**18**	Diseases of the male and female genital organs (including breast)	1,609 (1.9)	1,583 (98.4)	26 (1.6)
**19**	Benign neoplasms	1,550 (1.8)	1,522 (98.2)	28 (1.8)
**20**	Diseases of the esophagus, stomach and duodenum	1,541 (1.8)	1,487 (96.5)	54 (4.5)
	**Sum**	**62,116 (72.3)**	**59,377 (72.9)**	**2,739 (61.9)**
	**Other**	**23,788 (27.7)**	**22,100 (27.1)**	**1,688 (38.1)**
	**Total**	**85,904 (100)**	**81,477 (100)**	**4,127 (100)**

^a^ Diagnosis groups were classified first, according to ICD-10 codification and then re-grouped in broader categories (see methods and S1 and S2 Tables).

^b^ NLOS: Normal length-of-stay, PLOS: prolonged length-of-stay

**Fig 2:**
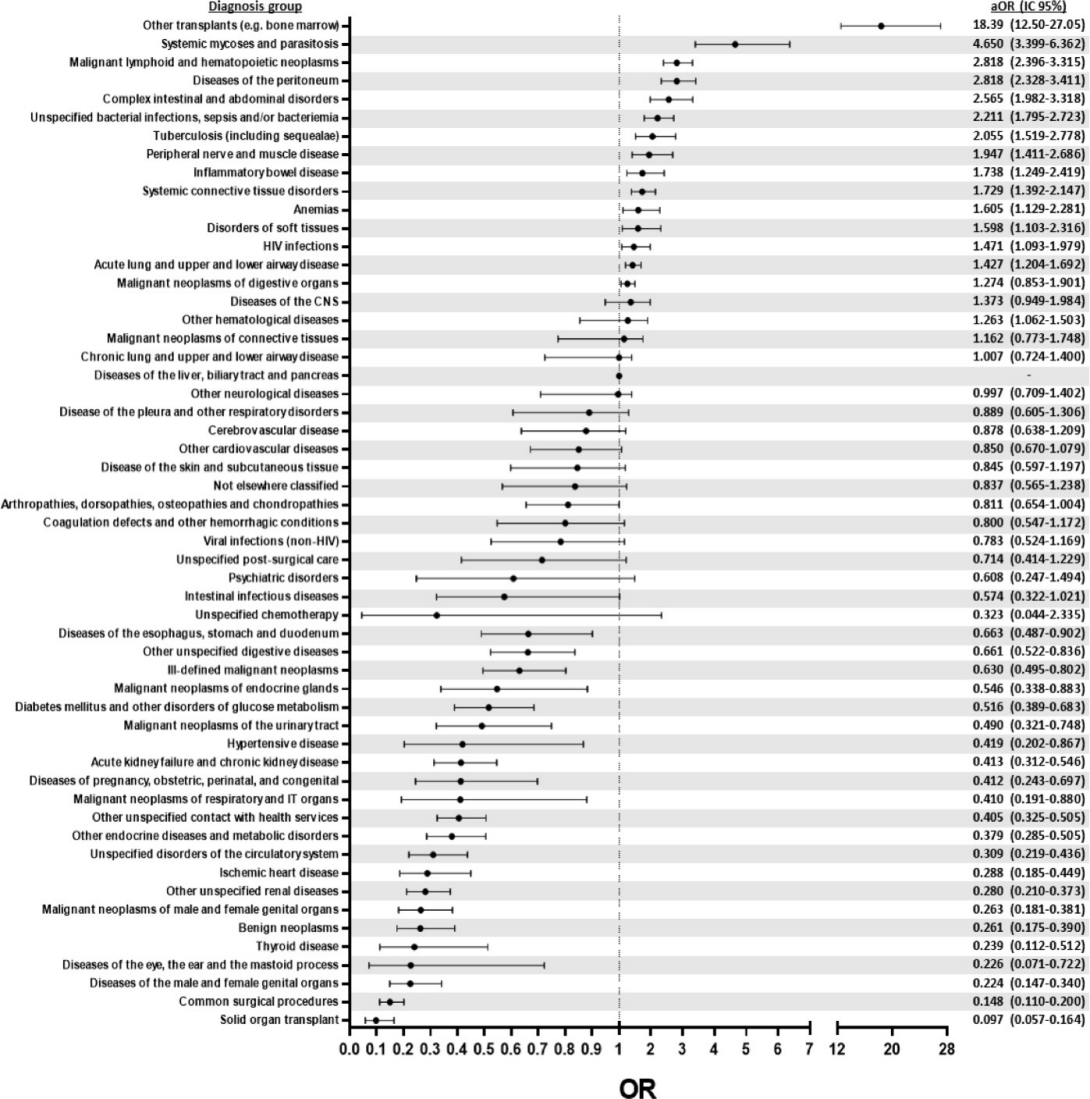
Adjusted risk of a prolonged length of stay (PLOS) event by diagnosis at hospital discharge. Odds ratios for PLOS by diagnosis at discharge were adjusted for age, gender, physician-to-patient ratio, type of admission, readmission at 30 days, day of admission (weekday vs weekend), number of additional diagnosis, place of residence and socioeconomic status using multinomial logistic regression models fixing “Diseases of the liver, biliary tract and pancreas (K70.0-K79.9, K83.0-K89.9)” as the reference group.

After adjusting for age, gender, physician-to-patient ratio, type of admission, readmission at 30 days, day of admission (weekday vs weekend), number of additional diagnosis, place of residence and socioeconomic status, we observed that hospitalization events associated to bone marrow transplant had the highest risk of PLOS (aOR = 18.4, 95% CI = 12.50–27.05); followed by systemic mycoses and parasitosis (aOR 4.6, 95% CI 3.9–6.4)(Fig 2). Hospitalizations events due to hematopoietic neoplasms (aOR 2.82, 95% CI 2.40–3.32), diseases of the peritoneum (aOR 2.82, 95% CI 2.33–3.41), complex intestinal and abdominal disorders (aOR 2.56, 95% CI 1.98–3.32), sepsis and severe bacterial infections (aOR 2.21, 95% CI 1.78–2.72), tuberculosis (aOR 2.05, 95% CI 1.52–2.78), peripheral nerve and muscle disorders (aOR 1.95, 95% CI 1.41–2.69), inflammatory bowel disease (aOR 1.74, 95%CI 1.25–2.15), among others also had an increased risk of PLOS (Fig 2). In contrast, hospitalizations for solid organ transplantation (aOR 0.10, 95% CI 0.06–0.16), due to common surgical procedures (aOR 0.15, 95% CI 0.11–0.20), disorders of the genital tract (aOR0.22, 95% CI 0.15–0.34) including neoplasms (aOR 0.26, 95% CI 0.17–0.39), thyroid disorders (aOR 0.24, 95% CI 0.11–0.51), among others were associated with a decreased risk of PLOS (Fig 2)

### Risk factors for PLOS

**Fig 3:**
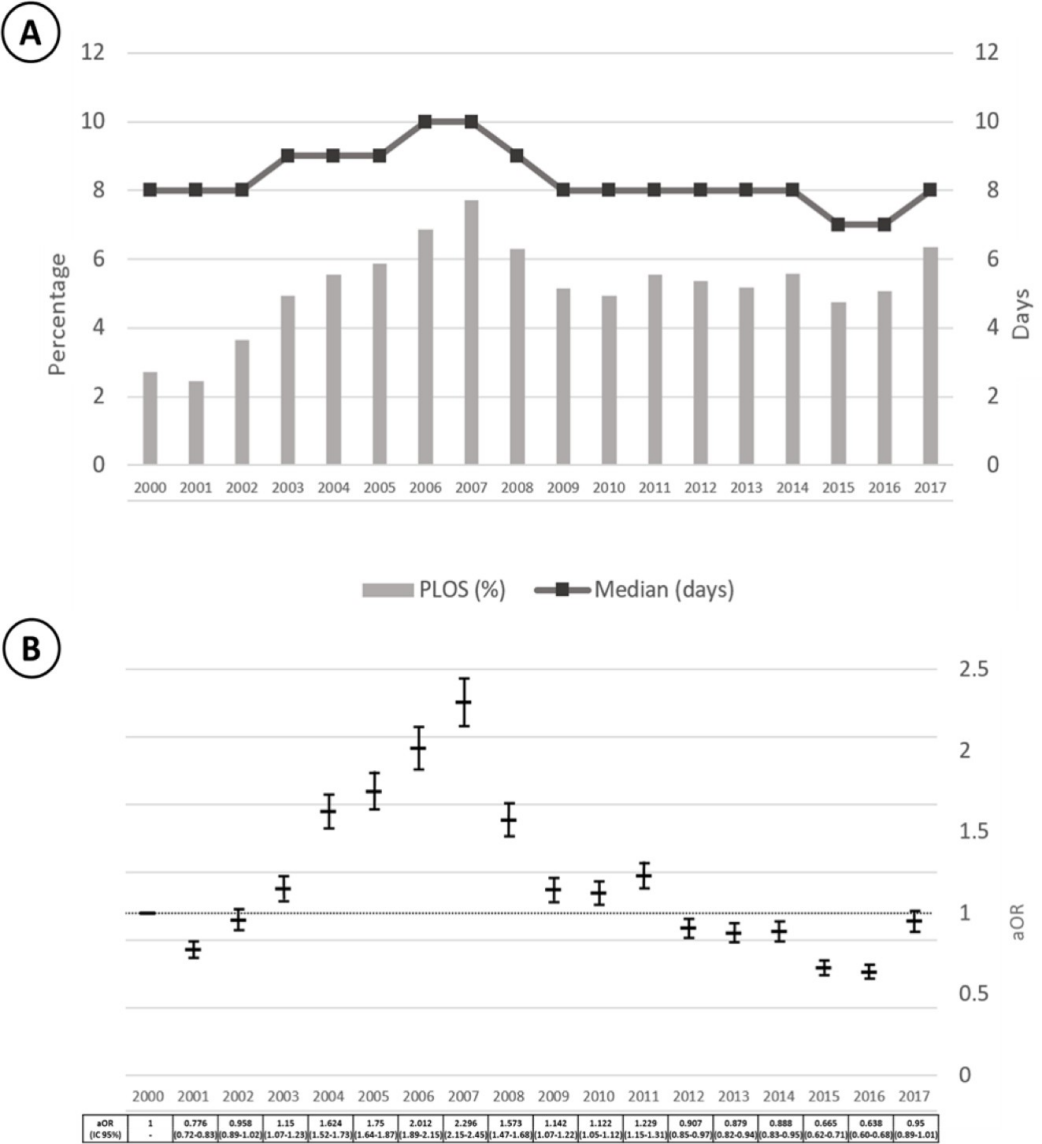
Annual frequency of hospitalizations classified as prolonged length-of-stay (PLOS) from 2000–2017. (A) The vertical, gray bars represent the annual percentage of hospitalization events classified as PLOS. The percentage increased from 2.4% in 2000 to 7.6% in 2007, then declined slightly in the ensuing years and remained stable during 2009–2016 with a later peak in 2017. The black, dotted line, summarizes the annual median length-of-stay (LOS) in days across time, during the study period. The median LOS for all hospitalization events was 8 days in 2000, peaked at 10 days in 2006 and 2007 and then declined to 8 days afterwards and up to 2015, when it declined again by one day (B). The black, vertical, boxplots illustrate the annual adjusted odds ratios (aORs) for prolonged stay of hospitalization (PLOS) using 2000 as the year of reference. We used multinomial logistic regression models to control for age, gender, type of admission, recent hospital discharge, weekday/weekend admission, additional diagnoses, place of residence and socioeconomic status, using inverse probability weights based on diagnosis of admission. The adjusted risk of PLOS increased between 2000 and 2007, then substantially and continuously decrease afterwards despite a sustained percentage of PLOS episodes after 2008.

After adjusting for the discharge diagnosis, we identified that age was independently, but weakly associated in an inverse manner with the risk of PLOS (1.2% risk reduction for each increasing year of age, 95%CI 1.1%-1.2%). Men had a slightly increased risk for PLOS than women (aOR 1.077, 95% CI 1.054–1.101), as well as early readmissions (aOR 1.05, 95% CI 1.02–1.09) and admission on weekends (Table 3). We also identified that emergency hospitalizations that required any type of surgical intervention ("emergency, surgical”) had the highest risk of PLOS in comparison to “elective, non-surgical” events of hospitalization (aOR 5.07, 95% CI 4.84–5.30). Moreover, there is an apparent, multiplicative interaction between urgent hospitalizations that require surgical interventions. Finally, having a low socioeconomic status also increased the risk of PLOS (Table 3). We also include the calendar-year in our model, and show the adjusted odds ratios for PLOS per calendar-year in Fig 3, Panel B, where we can observe that the adjusted risk of PLOS increases sharply between 2003 and 2007 in comparison to 2000, and then decreased afterwards in such degree of magnitude that the adjusted odds ratio of PLOS is lower in any calendar-year after 2012 in comparison to 2000. In addition, the unadjusted annual frequency of PLOS events and median LOS is summarized in Fig 3. Briefly, the crude frequency of PLOS increased between 2000 and 2007 from 2.4% to 7.6%, and then decreased the following three years to 5%, and appears to stabilize and vary between 5% to 6% until the end of the study.

**Table 3:** Multivariate analysis showing factors associated with prolonged length-of-stay (PLOS) from 2000–2017.

Variable	aOR^a^	IC 95%	P value
**Age**	0.988	0.988–0.989	<0.001
**Gender (male vs female)**	1.077	1.054–1.101	<0.001
**Type of admission (compared to elective, non-surgical)**			
• **Elective, surgical**	2.918	2.849–2.988	<0.001
• **Emergency, non-surgical**	1.803	1.731–1.877	<0.001
• **Emergency, surgical**	5.067	4.843–5.300	<0.001
**Hospitalization episode in the previous 30 days (compared to no recent hospitalization)**	1.052	1.018–1.088	0.003
**Weekday admission (compared to weekend)**	0.82	0.801–0.839	<0.001
**Additional diagnosis (for each additional diagnosis)**	1.313	1.307–1.319	<0.001
**Residence (within Mexico City)**	0.81	0.773–0.849	<0.001
**Socioeconomic status (compared to low socioeconomic level)**			
• **Middle**	0.594	0.580–0.609	<0.001
• **High**	0.722	0.698–0.748	<0.001

^a^ Adjusted using logistic regression model including age, gender, type of admission, year of admission (data shown in Fig 3), day of admission, additional diagnoses, place of residence and socioeconomic status using an inverse weight for the probability of PLOS by discharge diagnosis.

## Discussion

In this study, we analyzed >85,000 episodes of hospitalization in a tertiary healthcare referral center in Mexico City over a 18-year period, and we found that NLOS and PLOS patients are quite a distinct populations. We identified several risk factors for PLOS, that can be grouped as modifiable (perhaps preventable) and other non-modifiable, both recognizable at hospital admission. The modifiable risk factors include: physician-to-patient ratio and, potentially, the day of admission (weekday vs weekends). Among the non-modifiable risk factors, we found that younger age, male gender, type of admission and hospitalization (specially emergency and surgical admissions), the number of comorbidities, place of residence (outside of Mexico City) and a lower socioeconomic status were associated with an increased risk of PLOS. Also, certain diagnosis groups had an increased risk for PLOS, such as bone marrow transplant, fungal and bacterial infections, hematological neoplasms, complex intestinal and abdominal disorders, tuberculosis, and HIV-related infections, among others. Importantly, we also observed important changes in the frequency of PLOS over time and the adjusted risk of PLOS, during the study period, which are more noticeable before and after 2007, when the trend in increased frequency of PLOS and adjusted risk of PLOS over time, reversed significantly.

We hypothesize that changes in the infrastructure, organization and logistics in our hospital might account for these trends. While these, and other potentially unaccounted changes in organization occur gradually; we identified that increasing the physician-to-patient ratio, which occurred in February 2008, was associated to a decreased risk of PLOS. This change also led to a progressive decrease in the median LOS, which further supports the robustness of this measure. Information regarding physician-to-patient ratios and outcomes is scarce, but there is some evidence suggesting that increasing physician supply might reduce mortality and ED admissions [8]. On the other hand, to our knowledge this is the first study evaluating physician-to-patient ratio and its impact on LOS. Further investigations to identify other presently unaccounted changes in hospital infrastructure, organization and logistics are needed to better characterize this observation.

Weekend admissions, another potentially modifiable risk factor for PLOS, have already been associated with increased risk of PLOS and other poor outcomes (e.g. mortality). Still, factors leading to this “weekend effect” are not completely understood[9]. There are two potential explanations for this “weekend effect” at our institution: 1) Hospital staffing (physician to patient ratio) is lower on weekends and, 2) patients with complicated diseases referred from other hospitals (outside Mexico City) are admitted predominantly on weekends. Our study was not specifically designed to test these hypotheses, but further studies may confirm this finding.

Early readmissions (≤30 days), a measure of the quality of healthcare, are commonly considered as a risk factor for PLOS[10]. We observed that early readmission was associated with an increased risk of PLOS (aOR 1.05, 95% CI 1.018–1.088) when compared to late readmission (>30 days). Furthermore, we corroborated the phenomenon of increased risk of early readmission in the non-surgical population compared to the surgical population (32.1% vs 21.9%, *p<*0.001) that has already been reported in other studies [11, 12].

Surgical hospitalizations showed an increased risk for PLOS, both when elective (aOR 2.92, 95% CI 2.85–2.99) or emergency (aOR 5.07, 95% CI 4.84–5.30) admissions were considered. Surgical patients represent a considerable percentage of total hospitalizations events and risk factors for PLOS related to surgery have been previously described for several types of surgeries[13, 14]. This particular population should be further analyzed to dilucidate which specific factors of surgical interventions are associated with PLOS, which could aid in the design of preventive strategies for PLOS and other outcomes (e.g. mortality).

Other factors previously identified to be associated with PLOS are gender, hospitalizations in shared rooms, admissions through the ED, comorbidities and socioeconomic status. Hospitalizations in shared rooms have been reported to increase the risk of PLOS[15], but we did not observed this phenomenon in our population. Although an initial univariate analysis showed hospitalization in shared room to be a risk factor for PLOS, this effect did not persist after adjusting for socioeconomic level, which frequently determines the type of hospitalization room. Relationship between gender and risk of PLOS has been scarcely investigated, although female patients have been typically described as having more prolonged LOS [16, 17]. This contrasts with our results, and could be explained by a regional effect, as male patients in Mexico tend to seek less medical attention[18] which may translate in a more severe disease status at admission. We observed that patients residing outside Mexico City had an increased risk for PLOS. An explanation for this is difficult. However, patients referred from other hospitals (frequently outside Mexico City), with diseases that require more complex diagnostic workup or therapeutic approach. Admission through the ED increased the risk of PLOS in our study, as previously reported elsewhere[19, 20]. These patients are typically admitted in a more critical condition and have a high mortality; we found a mortality of 8.4% for those admitted through the ED vs 3.7% for those admitted directly to the wards (*p*<0.001). For those that survive their stay in the ED and are transferred to the general hospital wards, a more comprehensive diagnostic approach and therapeutic management is often required. This could explain the increased risk of PLOS in this population. The number of comorbidities and lower socioeconomic status were also associated with an increased risk of PLOS, as previously reported[21]. Finally, it is generally accepted that the leading determinant for a patient’s LOS is the main diagnosis. The binary logistic regression analysis of the discharge diagnoses demonstrated a predominance of complex diseases, such as hematological malignancies, complex infectious and intestinal/abdominal diseases and autoimmune diseases as risk factors for PLOS (Fig 2).

Even though PLOS patients typically represent a small percentage of the total population of hospitalized patients in other studies (3.6–5.6%)[22], they account for approximately 20% of hospital bed-days[6]. This is similar to our results (23.1% of total hospital bed-days). Considering that up to 44% of the health expenditure in Mexico is out-of-pocket, these numbers become extremely relevant due to the profound impact patients with PLOS have on the economy of their families, frequently leading to catastrophic health expenditure [23, 24]. PLOS were found to have a lower median socioeconomic status in our study, which further accentuates this issue. Finally, given that a significant proportion of patients (especially patients with PLOS) are not able to pay for their whole hospital stay, this also impacts the budget of the institution (in 2015, patients with PLOS at our Institution only covered 15.7% of their total hospitalization expenses; *unpublished data*).

PLOS has already been described as a risk factor for mortality, and previous studies in Mexico have reported that patients with a LOS >21 days have increased mortality (OR 2.41 [CI 95% 2.30–2.51])[25]. In our study, in-hospital crude mortality in patients with PLOS nearly tripled as compared to patients with NLOS. A lower mortality among patients with PLOS (4.4%) has been reported in other studies[20]. Our higher mortality could be explained, partly, by the fact that our study was conducted in a tertiary care referral hospital, included patients admitted from the ED and ICU and that we considered the 95^th^ percentile as a cut-off point to define PLOS. Further studies describing risk factors associated with mortality in our Institution are underway.

There are some limitations to our study. In particular, being a retrospective, cross-sectional analysis, our study is highly susceptible to different types of bias and confounding. While we used multivariate regression models to adjust for the potential confounding effect of measured variables, and use IPW to correct for the potential selection bias and confounding associated to discharge diagnosis, we may still have significant effect of unmeasured confounders. Also, patients receiving care at our institute are expected to have diseases of high-risk for PLOS; thus, it may be challenging to extrapolate our results to other medical institutions in our country. On the other hand, our study derives information from a real-world medical records database that is systematically populated since 2000, so we have consistent information about our hospital discharges for a considerable period of time. The database is completed by both physicians and professionally trained personnel, which decreases codification errors. Finally, our analyses are adjusted to multiple factors, which increases the robustness of our results.

## Conclusions

This is the first study analyzing risk factors for PLOS in Mexico and Latin America and herein we provide useful information from a large number of hospitalizations. We report some key sociodemographic and disease-specific differences in patients with PLOS which include a younger age, male gender, a lower physician-to-patient ratio, emergency and weekend admissions, surgery, the number of comorbidities, residence outside Mexico City and lower socioeconomic status. Importantly, we identified potentially modifiable factors associated with PLOS, which in our Institution appear to have contributed importantly to reduce LOS over time. We also observed several diagnosis groups to be associated with an increased risk for PLOS. Our findings could serve to develop a specific model of directed hospital healthcare once these factors are identified at admission and/or during hospitalization. Overall, this study provides data to guide research models that could culminate in public health policies to assess efficacy of healthcare at other public institutions and/or hospitals and prevent or correct risk factors for PLOS.

## Supporting information

S1 Table Discharge diagnosis groups. (DOCX)

S2 Table Types of surgeries. (DOCX)
