# Correction: Risk factors associated with prolonged hospital length-of-stay: 18-year retrospective study of hospitalizations in a tertiary healthcare center in Mexico

**DOI:** 10.1371/journal.pone.0209944

**Published:** 2018-12-21

**Authors:** The PLOS ONE Staff

In Table 1, the variable that reads "2 patients per medical team (March 2008-December 2017)" is incorrect. The variable should read "12 patients per medical team (March 2008-December 2017)". The publisher apologizes for the error.

**Table 1:** Comparison of characteristics of hospitalizations by type of episode (normal vs prolonged length-of-stay) from 2000–2017.

Characteristic	Normal length-of-stay (NLOS) (N = 81,477, 95%)	Prolonged length-of-stay (PLOS) (N = 4,427, 5%)	*p*
**Length-of-stay in days^a^**	8 (5–13)	45 (38–60)	*<*0.001
**Age at hospitalization**	52 (35–66)	48 (32–62)	<0.001
**Female (%)**	45,431 (55.76)	2,340 (52.9)	<0.001
**Residence (%)**			
• **Mexico City** • **Outside Mexico City** • **Unknown**	32,973 (40.5) 30,227 (37.1) 18,277 (22.4)	1,481 (33.5) 1,825 (41.2) 1,121 (25.3)	<0.001
**Type of admission (%)**			
• **Urgent** • **Elective**	8,939 (11.0) 72,538 (89.0)	1,277 (28.8) 3,150 (71.2)	<0.001
**Surgical patients (%)**	33,143 (40.7)	2,767 (62.5)	<0.001
**Shared Room (%)**	58,584 (71.9)	3,458 (78.1)	<0.001
**Patient to physician ratio (%)**			
• **20 patients per medical team (January 2000-February 2008)**	35,785 (43.9)	1,869 (42.2)	0.026
• **12 patients per medical team (March 2008-December 2017)**	45,692 (56.1)	2,558 (57.8)	
**Day of admission (%)**			
• **Weekday (Monday-Thursday)** • **Weekend (Friday-Sunday)**	56,329 (69.1) 25,148 (30.9)	2,842 (64.1) 1,591 (35.9)	<0.001
**Hospital readmissions (%)**	35,656 (43.8)	1,799 (40.6)	<0.001
**Distribution by type of readmission (%)**			
**• ≤ 30 days** **• >30 days**	9,937 (27.9) 25,719 (72.1)	605 (33.6) 1,194 (66.4)	<0.001
**Time for readmission**			
• **≤ 30 days** • **>30 days**	13 (7–21) 261 (93–793)	11 (6–18) 201 (81–713)	<0.001
**Surgeries per patient^b^**	1 (1)	2 (1–3)	<0.001
**Additional diagnoses**	3 (2–5)	5 (3–8)	<0.001
**Socioeconomic level**	3 (2–4)	2 (2–3)	<0.001
**Socioeconomic status^c^ (%)**			
• **Low (1–2)** • **Mid (3–4)** • **High (5–7)**	32,056 (39.3) 38,341 (47.1) 11,080 (13.6)	2,358 (53.3) 1,622 (36.6) 447 (10.1)	<0.001
**In-hospital Mortality (%)**	3,035 (3.7)	588 (13.3)	<0.001
**Location of death (%)**			
• **Hospital ward** • **Emergency Department** • **Intensive care unit**	1,887 (62.2) 574 (18.9) 574 (18.9)	272 (46.3) 110 (18.7) 206 (35.0)	<0.001

^a^ Continuous variables are summarized using medians and interquartile ranges (IQR)

^b^ Only surgical patients were included in this analysis (N = 35,910)

^c^ Socioeconomic status is a construct used by the MNIH that comprises the following elements: monthly household income, family`s main provider`s occupation, monthly household expenses, type of housing and family`s health status. Patients are classified in seven levels (1–7) and that determines the amount the patient should pay for healthcare.
