# Supplementary material for: Risk factors associated with prolonged hospital length-of-stay: 18-year retrospective study of hospitalizations in a tertiary healthcare center in Mexico
Source: PLoS One. 2018 Nov 8;13(11):e0207203. doi: 10.1371/journal.pone.0207203 (PMC6224124; doi:10.1371/journal.pone.0207203)
Supplement: S1 Table — (DOCX) [file pone.0207203.s001.docx]

**S1 Table.** Discharge diagnosis groups

|  | ICD-10 CODES | DIAGNOSIS GROUP | ALL (% OF THE COLUMN) | NLOS (% OF THE ROW) | PLOS (% OF THE ROW) |
| --- | --- | --- | --- | --- | --- |
| 1 | A00.0-A09.9 | Intestinal infectious diseases | **572 (0.7)** | **558 (97.6)** | **14 (2.4)** |
| 2 | A10.0-A19.9, B90.0-B90.9 | Tuberculosis (including sequealae) | **564 (0.7)** | **492 (87.2)** | **72 (12.8)** |
| 3 | A20.0-A79.9, B92.0-B96.9, B99.0-B99.9 | Unspecified bacterial infections, sepsis and/or bacteriemia | **1,449 (1.7)** | **1,247 (86.1)** | **202 (13.9)** |
| 4 | A80.0-B19.9, B25.0-B34.9, B91.0-B91.9, B97.0-B98.9 | Viral infections (non-HIV) | **923 (1.1)** | **892 (96.6)** | **31 (3.4)** |
| 5 | B20.0-B24.9 | HIV infections | **835 (1.0)** | **760 (91.0)** | **75 (9.0)** |
| 6 | B35.0-B89.9 | Systemic mycoses and parasitosis | **377 (0.4)** | **292 (77.5)** | **85 (22.5)** |
| 7 | C00.0-C29.9, D00.0-D01.9 | Malignant neoplasms of digestive organs (oral cavity to anus) | **5,517 (6.4)** | **5,214 (94.5)** | **303 (5.5)** |
| 8 | C30.0-C39.9, D02.0-D02.9 | Malignant neoplasms of respiratory and intrathoracic organs | **434 (0.5)** | **424 (97.7)** | **10 (2.3)** |
| 9 | D40.0-C49.9, D03.0-D04.9 | Malignant neoplasms of bone, articular cartilage, skin, mesothelial and soft tissues | **553 (0.6)** | **524 (94.8)** | **29 (5.2)** |
| 10 | C50.0-C63.9, D05.0-D08.9 | Malignant neoplasms of male and female genital organs (including breast) | **2,621 (3.1)** | **2,585 (98.6)** | **36 (1.4)** |
| 11 | C64.0-C68.9 | Malignant neoplasms of the urinary tract | **935 (1.1)** | **910 (97.3)** | **25 (2.7)** |
| 12 | C73.0-C75.9 | Malignant neoplasms of the thyroid and other endocrine glands | **619 (0.7)** | **599 (96.8)** | **20 (3.2)** |
| 13 | C76.0-C80.9, C97.0-C99.9, D09.0-D09.9, D37.0-D49.9 | Ill-defined, secondary and of uncertain behavior malignant neoplasms | **3,213 (3.7)** | **3,112 (96.9)** | **101 (3.1)** |
| 14 | C81.0-C96.9 | Malignant neoplasms of lymphoid, hematopoietic and related tissue | **6,602 (7.7)** | **6,127 (92.8)** | **475 (7.2)** |
| 15 | D10.0-D36.9 | Benign neoplasms | **1,550 (1.8)** | **1,522 (98.2)** | **28 (1.8)** |
| 16 | D50.0-D64.9 | Anemias (including aplastic, hemolytic, etc.) | **805 (0.9)** | **760 (94.4)** | **45 (5.6)** |
| 17 | D65.0-D69.9 | Coagulation defects, purpura and other hemorrhagic conditions | **915 (1.1)** | **881 (96.3)** | **34 (3.7)** |
| 18 | D70.0-D89.9 | Other hematological diseases (including agranulocitosis/neutropenia, and immune disorders) | **580 (0.7)** | **547 (94.3)** | **33 (5.7)** |
| 19 | E00.0-E09.9 | Thyroid disease | **612 (0.7)** | **604 (98.7)** | **8 (1.3)** |
| 20 | E10.0-E16.9 | Diabetes mellitus and other disorders of glucose metabolism | **1,696 (2.0)** | **1,635 (96.4)** | **61 (3.6)** |
| 21 | E20.0-E99.9 | Other endocrine diseases and metabolic disorders (e.g. obesity, dyslipidemia, malnutrition, etc.) | **2,235 (2.6)** | **2,167 (97.0)** | **68 (3.0)** |
| 22 | F00.0-F99.9 | Psychiatric disorders | **386 (0.4)** | **378 (97.9)** | **8 (2.1)** |
| 23 | C69.0-C72.9, G00.0-G34.9 | Infectious, inflammatory, degenerative, and neoplastics diseases of the CNS | **584 (0.7)** | **540 (92.5)** | **44 (7.5)** |
| 24 | G45.0-G46.9, I60.0-I69.9 | Cerebrovascular disease | **899 (1.0)** | **843 (93.8)** | **56 (6.2)** |
| 25 | G35.0-G44.9, G47.0-G49.9, G80.0-G99.9 | Other neurological diseases | **804 (0.9)** | **757 (94.2)** | **47 (5.8)** |
| 26 | G50.0-G799 | Peripheral nerve and muscle disease | **637 (0.7)** | **585 (91.8)** | **52 (8.2)** |
| 27 | H00.0-H99.9 | Diseases of the eye, the ear and the mastoid process | **239 (0.3)** | **236 (98.7)** | **3 (1.3)** |
| 28 | I10.0-I19.9 | Hypertensive disease | **359 (0.4)** | **351 (97.8)** | **8 (2.2)** |
| 29 | I20.0-I25.9 | Ischemic heart disease | **1,058 (1.2)** | **1,035 (97.8)** | **23 (2.2)** |
| 30 | I00.0-I09.9, I26.0-I59.9, I90.0-I99.9 | Other cardiovascular diseases (including rheumatic and pulmonary heart disease) | **1,852 (2.2)** | **1,737 (93.8)** | **115 (6.2)** |
| 31 | I70.0-I89.9 | Unspecified disorders of the circulatory system | **1,641 (1.9)** | **1,599 (97.4)** | **42 (2.6)** |
| 32 | J00.0-J29.9, J69.0, J80.0-J82.9, J85.0-J89.9, J96.0-J97.9 | Acute lung and upper and lower airway disease | **4,561 (5.3)** | **4,185 (91.8)** | **376 (8.2)** |
| 33 | J30.0-J68.9, J69.1-J79.9, J83.0-J84.9 | Chronic lung and upper and lower airway disease | **807 (0.9)** | **762 (94.4)** | **45 (5.6)** |
| 34 | J90.0-J95.9, J98.0-J99.9 | Disease of the pleura and other respiratory disorders | **626 (0.7)** | **593 (94.7)** | **33 (5.3)** |
| 35 | K00.0-K34.9 | Diseases of the esophagus, stomach and duodenum | **1,541 (1.8)** | **1,487 (96.5)** | **54 (4.5)** |
| 36 | K35.0-K49.9, K80.0-K82.9 | Common surgical procedures (appendectomy, hernia repair, cholecystectomy) | **3,871 (4.5)** | **3,815 (98.6)** | **56 (2.4)** |
| 37 | K50.0-K54.9 | Inflammatory bowel disease | **786 (0.9)** | **738 (93.9)** | **48 (6.1)** |
| 38 | K55.0, K56.1-K56.2, K56.5, K57.0, K57.2, K57.4, K57.8, K63.0-K63.4 | Complex intestinal and abdominal disorders (e.g. intestinal fistula) | **535 (0.6)** | **414 (77.4)** | **121 (22.6)** |
| 39 | K55.1-K56.0, K56.3-K56.4, K56.6-K56.7, K57.1, K57.9, K57.3,  K57.5, K58.0-K62.9, K63.5-K64.9, K90.0-K99.9 | Other unspecified digestive diseases (including intestinal obstruction, functional disorders, GI bleeding, etc.) | **2,655 (3.1)** | **2,547 (95.9)** | **108 (4.1)** |
| 40 | K65.0-K69.9 | Diseases of the peritoneum (e.g. peritonitis) | **1,521 (1.8)** | **1,231 (80.9)** | **290 (19.1)** |
| 41 | L00.0-L99.9 | Disease of the skin and subcutaneous tissue (e.g. cellulitis) | **994 (1.2)** | **951 (95.7)** | **43 (4.3)** |
| 42 | M00.0-M29.9, M40.0-M59.9, M80.0-M99.9 | Arthropathies, dorsopathies, osteopathies and chondropathies | **2,978 (3.5)** | **2,829 (95.0)** | **149 (5.0)** |
| 43 | M30.0-M39.9 | Systemic connective tissue disorders | **1,858 (2.2)** | **1,704 (91.7)** | **154 (8.3)** |
| 44 | M60.0-M79.9 | Disorders of the muscles, synovium, tendons, and other soft tissues | **427 (0.5)** | **386 (90.4)** | **41 (9.6)** |
| 45 | N17.0-N19.9 | Acute kidney failure and chronic kidney disease | **1,904 (2.2)** | **1,840 (96.6)** | **64 (3.4)** |
| 46 | N00.0-N16.9, N20.0-N39.9, N99.0-N99.9 | Other unspecified renal diseases (e.g. glomerular, interstitial, etc.) | **3,999 (4.7)** | **3,935 (98.4)** | **64 (1.6)** |
| 47 | N40.0-N98.9 | Diseases of the male and female genital organs (including breast) | **1,609 (1.9)** | **1,583 (98.4)** | **26 (1.6)** |
| 48 | O00.0-Q99.9 | Diseases of pregnancy, obstetric, perinatal, and congenital | **513 (0.6)** | **495 (96.5)** | **18 (3.5)** |
| 49 | R95.0-Z39.9, Z41.0-Z41.9, Z43.0-Z46.9, Z49.0-Z51.0, Z51.3-Z53.9, Z55.0-Z79.9, Z95.0-Z97.9, Z99.0-Z99.9, Z80.0-Z93.9, Z40.0-Z40.9 | Other unspecified healthcare and contact with health services (e.g. trauma, burns, dependency on ventilators and other devices, etc.) | **3,787 (4.4)** | **3,657 (96.6)** | **130 (3.4)** |
| 50 | Z51.1-Z51.2 | Unspecified chemotherapy | **182 (0.2)** | **181 (99.5)** | **1 (0.5)** |
| 51 | Z42.0-Z42.9, Z47.0-Z48.9, Z54.0-Z54.9, Z98.0-Z98.9 | Unspecified post-surgical care | **216 (0.3)** | **198 (91.7)** | **18 (8.3)** |
| 52 | Z94.0-Z94.7 | Solid organ transplant | **942 (1.1)** | **922 (97.9)** | **20 (2.1)** |
| 53 | Z94.8-Z94.9 | Other transplants (e.g. bone marrow) | **182 (0.2)** | **130 (71.4)** | **52 (18.6)** |
| 54 | R00.0-R94.9 | Symptoms, signs and abnormal clinical and laboratory findings, not elsewhere classified | **918 (1.1)** | **884 (96.3)** | **34 (3.7)** |
| 55* | K70.0-K79.9, K83.0-K89.9 | Diseases of the liver, biliary tract and pancreas | **6,426 (7.5)** | **6,097 (94.9)** | **329 (5.1)** |
|  |  | **TOTAL** | **85,904 (100.0)** | **81,477 (94.8)** | **4,427 (5.2)** |

* Reference category for the binary logistic regression analysis
