# Supplementary material for: Risk factors associated with prolonged hospital length-of-stay: 18-year retrospective study of hospitalizations in a tertiary healthcare center in Mexico
Source: PLoS One. 2018 Nov 8;13(11):e0207203. doi: 10.1371/journal.pone.0207203 (PMC6224124; doi:10.1371/journal.pone.0207203)
Supplement: S2 Table — (DOCX) [file pone.0207203.s002.docx]

**S2 Table.** Types of surgeries

|  | ICD-9 CODES | TYPE OF SURGERY^*^ | ALL (% OF COLUMN) | NLOS (% OF ROW) | PLOS (% OF ROW) |
| --- | --- | --- | --- | --- | --- |
| 1 | 00.91-00.93, 50.50-50.59, 55.60-55.69 | Transplant surgery | **1,709 (3.3)** | **1,635 (95.7)** | **74 (4.3)** |
| 2 | 01.00-05.99, 07.60-07.89 | Operations on the nervous system (including the pituitary) | **1,863 (3.6)** | **1,623 (87.1)** | **240 (12.9)** |
| 3 | 06.00-06.99 | Operations on the thyroid and parathyroid glands | **1,277 (2.5)** | **1,222 (95.7)** | **55 (4.3)** |
| 4 | 07.00-07.59, 07.90-07.99 | Operations on other endocrine glands | **291 (0.6)** | **268 (92.1)** | **23 (7.9)** |
| 5 | 08.00-29.99 | Operations on the eye, ear, nose, mouth, and pharynx | **588 (1.1)** | **533 (90.6)** | **55 (9.4)** |
| 6 | 30.00-31.99 | Operations on the upper airways | **179 (0.3)** | **134 (74.9)** | **45 (25.1)** |
| 7 | 32.00-34.99 | Operations on the lower airways | **2,075 (4.0)** | **1,575 (75.9)** | **500 (24.1)** |
| 8 | 35.00-37.99 | Operations on the heart (including valves and pericardium) | **1,031 (3.0)** | **856 (83.0)** | **175 (17.0)** |
| 9 | 38.00-39.99 | Unspecified vascular surgery | **2,642 (5.1)** | **2,414 (91.4)** | **228 (8.6)** |
| 10 | 40.00-40.99 | Operations on the lymphatic system | **1,149 (2.2)** | **1,083 (94.3)** | **66 (5.7)** |
| 11 | 41.00-41.99 | Operations on the bone marrow and spleen | **830 (1.6)** | **728 (87.7)** | **102 (12.3)** |
| 12 | 42.00-42.99 | Operations on the esophagus | **840 (1.6)** | **690 (82.1)** | **150 (17.9)** |
| 13 | 43.00-44.99 | Operations on the stomach | **2,166 (4.2)** | **1,888 (87.2)** | **278 (12.8)** |
| 14 | 45.00-46.99 | Operations on the small bowel | **6,138 (11.8)** | **5,020 (81.8)** | **1,118 (19.2)** |
| 15 | 47.00-47.99, 51.20-51.29, 53.00-53.99 | Common general surgery operations (appendectomy, hernia repair and cholecystectomy) | **4,597 (8.9)** | **4,377 (95.2)** | **220 (4.8)** |
| 16 | 48.00-49.99 | Operations on the large intestine, rectum and the anus | **912 (1.8)** | **845 (92.7)** | **67 (7.3)** |
| 17 | 50.00-50.49, 50.60-51.19, 51.30-51.99 | Operations on the liver and biliary tract (excluding transplant and cholecystectomy) | **3,445 (6.6)** | **3,046 (88.4)** | **399 (11.6)** |
| 18 | 52.00-52.99 | Operations on the pancreas | **1,207 (2.3)** | **902 (74.7)** | **305 (25.3)** |
| 19 | 54.00-54.99 | Other abdominal operations (including unspecified laparotomy, peritoneal lavage, etc.) | **6,078 (11.7)** | **4,836 (79.6)** | **1,242 (20.4)** |
| 20 | 55.00-55.59, 55.70-55.99 | Operations on the kidney (excluding transplant) | **2,067 (4.0)** | **1,989 (96.2)** | **78 (3.8)** |
| 21 | 56.00-59.99 | Operations on the urinary tract and urinary bladder) | **1,153 (2.2)** | **1,103 (95.7)** | **50 (4.3)** |
| 22 | 60.00-71.99 | Operations on the male and female genital organs | **3,589 (6.9)** | **3,485 (97.1)** | **104 (2.9)** |
| 23 | 72.00-75.99 | Obstetric procedures | **27 (0.1)** | **27 (100.0)** | **0 (0)** |
| 24 | 76.00-78.99 | Unspecified incision, excision and division of the bone | **427 (0.8)** | **371 (86.9)** | **56 (13.1)** |
| 25 | 79.00-79.99 | Fracture reduction and dislocation | **74 (0.1)** | **69 (93.2)** | **5 (6.8)** |
| 26 | 80.00-80.99 | Incision and excision of the joint structures | **537 (1.0)** | **432 (80.4)** | **105 (10.6)** |
| 27 | 70.89, 81.00-81.99 | Repair and plastic operations on joint structures | **1,703 (3.3)** | **1,661 (97.5)** | **42 (2.5)** |
| 28 | 82.00-8399 | Operations on muscle, tendon and fascia | **640 (1.2)** | **555 (86.7)** | **85 (13.3)** |
| 29 | 84.00-8499 | Other unspecified operations on the musculoskeletal system | **958 (1.8)** | **846 (88.3)** | **112 (11.7)** |
| 30 | 85.00-8699 | Operations on the breast, skin and subcutaneous tissue | **1,732 (3.3)** | **1,518 (87.6)** | **214 (12.4)** |
|  |  | **TOTAL** | **51,924 (100)** | **45,731 (88.1)** | **6,193 (11.9)** |

* Procedures not performed in the operation room were manually excluded (e.g. 87.00-99.99, among others)
